# Measuring Impatience in Intertemporal Choice

**DOI:** 10.1371/journal.pone.0149256

**Published:** 2016-02-18

**Authors:** Salvador Cruz Rambaud, María José Muñoz Torrecillas

**Affiliations:** Departamento de Economía y Empresa, Facultad de Ciencias Económicas y Empresariales, Universidad de Almería, La Cañada de San Urbano, s/n, 04120, Almería, Spain; Middlesex University London, UNITED KINGDOM

## Abstract

In general terms, decreasing impatience means decreasing discount rates. This property has been usually referred to as hyperbolic discounting, although there are other discount functions which also exhibit decreasing discount rates. This paper focuses on the measurement of the impatience associated with a discount function with the aim of establishing a methodology to compare this characteristic for two different discount functions. In this way, first we define the patience associated with a discount function in an interval as its corresponding discount factor and consequently we deduce that the impatience at a given moment is the corresponding instantaneous discount rate. Second we compare the degree of impatience of discount functions belonging to the same or different families, by considering the cases in which the functions do or do not intersect.

## Introduction

*Impatience* was already defined in 1960 by [1] as the decrease in the aggregate utility with respect to time. In his work, he stated: “this study started out as an attempt to formulate postulates permitting a sharp definition of impatience, the short term Irving Fisher has introduced for preference for advanced timing of satisfaction” ([1] referred to the 1930 work of Fisher [2]: “The Theory of Interest” (Chapter IV)). This idea of a preference for advancing the timing of future satisfaction has been used in economics since the appearance of Böhm-Bawerk’s work: *Positive Theorie des Kapitals* [3].

Some authors use the term *impulsivity* as a synonym of impatience, e.g. [4]. In effect, [5] define impulsivity in intertemporal choice as a “strong preference for small immediate rewards over large delayed ones”. We can find a similar and earlier definition in [6] who defined the impulsiveness (in choices among outcomes of behavior) as “the choice of the less rewarding over more rewarding alternatives”. Observe that impatience has usually been presented in relative terms by comparing the values shown by two intertemporal choices. [7] states that the term impulsivity is often utilized in psychiatric studies on intertemporal choice and cites some examples of impulsive subjects such as smokers, addicts and attention-deficient hyperactivity-disorder patients. The opposite behavior to impulsivity is self-control.

As for the measure of impatience, the rate of discount is commonly taken to indicate the level of impulsivity or impatience in intertemporal choices. [8] offer an interesting review of the empirical research on intertemporal choice and summarize the implicit discount rates from all the studies they reviewed. In the same way, [9] and [10] provide a revision on time-declining discount rates from the observed individual choice, among other approaches.

But, as will be demonstrated in the next section, there are other ways to quantify the impatience. Our main objective in this paper is to develop a measure of the impatience exhibited by the discount function associated with the underlying intertemporal choice. In this case, we will be able to compare the impatience associated with two discount functions.

On this subject, there are many empirical papers trying to compare the degree of impatience of a group of individuals at different points in time (e.g. [11], [12], [13], [14]) or to compare the degree of impatience with different discount functions (e.g. [7], [13], [15], [16], [17], [18]). There is also an alternative measure of discounting: the area under the curve proposed by [19], which allows comparing the impatience between individuals in a model-free way (since it is not tied to any specific theoretical framework). See [20] for a review of this method and reference to several studies in which it has been employed.

Another approach related to this topic is the analysis of the main types of impatience. Thus, when studying the impatience in intertemporal choice, we usually find that it decreases. Following [21], *decreasing impatience* implies an inverse relationship between the discount rate and the magnitude of the delay and has usually been attributed to *hyperbolic discounting*. In the same way, [22] treats decreasing impatience as the core property which is parametrically expressed by hyperbolic and quasi-hyperbolic discount functions.

Recently, several studies have included different degrees of impatience and not only decreasing impatience ([23], [24], [25], [26], [27], [28]). [25] report individual evidence of lower discounting for intervals closer to the present than for distant ones, demonstrating concave discounting, which implies *increasing impatience*.

Additionally, a number of empirical papers have recently appeared which relate impatience with decision-making in games. For example, [29] study the relationship between impatience, risk aversion, and household income. [30] conducts a research on impatience, risk aversion, and working environment. [31] empirically study the patience/impatience of punishers in a multilateral cooperation game. In a similar vein, [32] explore the relationship between impatience and bargaining behavior in the ultimatum game.

In this paper the concept of *patience* associated with a discount function (*F*(*t*)) in an interval [*t*_1_, *t*_2_] is defined as the value of the discount factor corresponding to *F*(*t*) in this interval. Hence, the impatience associated with *F*(*t*) in an interval will be calculated as 1 minus the discount factor associated with the given interval, which is the value of the discount corresponding to $1 in this interval. Additionally, we present a procedure to compare the degree of impatience between two discount functions, of the same or different family. In [33] we find a comparison between exponential and hyperbolic discount functions controlling the overall impatience in order to isolate the differences due to self-control problems only. The controlled comparison is made by means of age adjustment which equalizes areas under discount functions.

The objective of this paper is interesting for the following reasons:

1. First of all, fitting the preferences exhibited by an individual or a group of individuals to a well-known discount function has an important advantage. In effect, the questionnaires used in intertemporal choice include a limited number of pairs of amounts and delays. Nevertheless, a discount function fitted to data from respondents allows us to analyze the preferences between any pair of monetary rewards.
2. Inevitably, most researches on this issue show the discount functions which, in each case, better fit the data. In effect, [34] estimate the parameters of the main intertemporal choice models: exponential, simple hyperbolic, quasi-hyperbolic, and *q*-exponential. Subsequently, they compare the impatience shown by two groups by simply comparing the discount rates of the corresponding discount functions. Obviously, this is not an accurate procedure because some discount functions are biparametric and so it should require a comparison of both parameters defining the function. Moreover, this is a simplification because it would be interesting to compare the impatience in a certain time interval where, among other circumstances, the relative position of the impatience levels can change. Even the use of the *q*-exponential discount function assumes working with an exponential, a hyperbolic or a generalized hyperbolic discount function, depending on the concrete values of *q* and *k*_*q*_. Therefore, the most important thing is to obtain the discount function which better fits the collected data, and then it is likely that the subsequent comparison can involve discount functions belonging to different families. Even the comparison between two discount functions belonging to the same family (for instance, two hyperbolic discount functions) is also noteworthy because they usually exhibit different parameters.
3. Several researches have considered the impatience shown by individuals of different nationalities, genders or socio-economic levels. The comparison of the discount functions involved in these studies is important in order to design, for example, a market segmentation strategy according to the former criteria.
  [35] state that the intertemporal impatience can be applied to the acquisition of material objects instead of money. This makes the issue of impatience very interesting in marketing and consumer behavior. They point out some culture-related differences between western and eastern participants in the empirical study conducted by them: the former valued immediate consumption more than the latter. In the same way, [14] experimentally compared intertemporal choices for monetary gains and losses by American and Japanese subjects, demonstrating that Westerners are more impulsive and time-inconsistent than Easterners. [36] also recognize the accuracy of discounting to explain impatience in marketing.
  Finally, [37] have found that gender and autobiographical memory can have an effect on delay discounting: there is a significant difference between men and women because, in the case of higher memory scores, the former showed less impatience when discounting future rewards. In the experimental analysis, they used the standard hyperbolic and the quasi-hyperbolic models.
  It is therefore apparent that the comparison of discount functions will be of interest to segment a market depending on the impatience exhibited by individuals who are classified by different criteria (geographical, gender, culture, etc.).

This paper is organized as follows. After this introduction, in Section 2 we will formally define the impatience (impulsivity) ranging from the discount corresponding to $1 in an interval [*t*_1_, *t*_2_] (a two-parameter function, referred to as *impatience-arc*) to the instantaneous discount rate at an instant *t* (a one-parameter function, referred to as *instantaneous impatience*). The value of the instantaneous rate at *t* = 0 (a constant) can also be taken into account. Obviously, any simplification in the measurement of impatience will result in a reduction in the amount of information thus obtained. Therefore, in Section 3, we will compare the impatience associated with two discount functions, considering two cases: when the functions do not intersect and the functions do intersect. In Section 4, all the obtained results will be applied to well-known families of discount functions. Finally, Section 5 summarizes and concludes.

## Defining impatience (impulsivity) in intertemporal choice

In economics and other social sciences it is common practice to try to simplify the complexity of the models describing the behavior corresponding to a group of people. This is the case of discount functions in the framework of intertemporal choice within the field of finance. In effect, a (dynamic) intertemporal choice can be described by a *two-variable discount function* ([38]), that is, a continuous function

$$\begin{array}{c} F:R\times R^{+}⟶R \end{array}$$

such that

$$\begin{array}{c} (d,t)\mapsto F(d,t), \end{array}$$

where *F*(*d*, *t*) represents the value at *d* (*delay*) of a $1 reward available at instant *d* + *t*. In order to make financial sense, this function must satisfy the following *conditions*:

1. *F*(*d*, 0) = 1,
2. *F*(*d*, *t*) > 0, and
3. For every *d*, *F*(*d*, *t*) is strictly decreasing with respect to *t*.

A discount function is said to be

1. *With bounded domain* if, for every d∈R, there exists an instant $t_{d}\in R^{+}$, depending on *d*, such that *F*(*d*, *t*_*d*_) = 0.
2. *With unbounded domain* if, for every d∈R and $t\in R^{+}$, one has *F*(*d*, *t*) > 0. Within this group, a discount function can be:
  - *Regular* if $\underset{t\to +\infty }{\text{lim}}F(d,t)=0$, for every d∈R.
  - *Singular* if $\underset{t\to +\infty }{\text{lim}}F(d,t)>0$, for every d∈R.

Regular discount functions are the most usual valuation financial tools. Nevertheless, and as indicated at the beginning of this Section, this discounting model can be simplified by using a function *F*(*t*) independent of delay *d*. More specifically, a *one-variable discount function* *F*(*t*) ([38] and [39]) is a continuous real function

$$\begin{array}{c} F:R^{+}⟶R \end{array}$$

such that

$$\begin{array}{c} t\mapsto F(t), \end{array}$$

defined within an interval [0, *t*_0_) (*t*_0_ can even be +∞), where *F*(*t*) represents the value at 0 of a $1 reward available at instant *t*, satisfying the following conditions:

1. *F*(0) = 1,
2. *F*(*t*) > 0, and
3. *F*(*t*) is strictly decreasing.

The following theorem of representation provides the relationship between the preferences existing in a scenario of intertemporal choice and its associated discount function.

**Theorem 1**. A discount function *F*(*t*) gives rise to the total preorder ≽ defined by

$$\begin{array}{c} \left(C_{1},t_{1}\right)⪰\left(C_{2},t_{2}\right)\;\text{if}\;C_{1}F\left(t_{1}\right)\geq C_{2}F\left(t_{2}\right), \end{array}$$

satisfying the following conditions:

1. If *t*_1_ ≤ *t*_2_, then (*C*, *t*_1_) ≽ (*C*, *t*_2_), and
2. If *C*_1_ ≥ *C*_2_, then (*C*_1_, *t*) ≽ (*C*_2_, *t*).

Reciprocally, every total preorder ≽ satisfying conditions (i) and (ii) defines a discount function.

Theorem 1 shows that, in intertemporal choice, an agent can indistinctly use a discount function or a total preorder. In this way, the concept of impatience has been mainly treated with a total preorder. For example, [40] propose the following choice: “$10 in a year or $15 in a year and a week”. In this way, they state that: “If an individual A prefers the first option ($10 in a year) while B prefers the second option ($15 in a year and a week), it is said that A is more impulsive than B because A prefers a smaller, but more immediate reward, whereas B prefers to wait a longer time interval to receive a greater reward”. Nevertheless, our aim here is to define the concept of impatience by using discount functions. In effect, given a one-variable discount function *F*(*t*), the *patience* associated with *F*(*t*) in an interval [*t*_1_, *t*_2_] (*t*_1_ < *t*_2_) is defined as the value of the discount factor *f*(*t*_1_, *t*_2_) corresponding to this interval, viz:

$$\begin{array}{c} f\left(t_{1},t_{2}\right):=\frac{F(t_{2})}{F(t_{1})}=\exp\{-\int_{t_{1}}^{t_{2}}\delta \left(x\right)\text{d}x\}, \end{array}$$ (1)

where $\delta \left(x\right)={-\frac{\text{d}\ln F(z)}{\text{d}z}|}_{z=x}$ is the instantaneous discount rate of *F*(*t*) at instant *x*. Obviously, the inequality 0 < *f*(*t*_1_, *t*_2_) < 1 holds. Observe that the greater the discount factor, the less sloped is the discount function in the interval [*t*_1_, *t*_2_]. In this case, people are willing to wait for a long time to receive a future amount because they have to renounce a small part of their money. On the other hand, the *impatience* associated with *F*(*t*) in the interval [*t*_1_, *t*_2_] (*t*_1_ < *t*_2_) is defined as the value of the discount *D*(*t*_1_, *t*_2_) corresponding to this interval, viz:

$$\begin{array}{c} D\left(t_{1},t_{2}\right):=1-f\left(t_{1},t_{2}\right), \end{array}$$ (2)

which lies in the interval [0, 1].

Some comments:

1. It is logical that the impatience can be measured by the amount of money that the agent is willing to lose in exchange for anticipating the availability of a $1 reward.
2. Any function with the same monotonicity as *f*(*t*_1_, *t*_2_) (resp. *D*(*t*_1_, *t*_2_)) can be used as a measure of patience (resp. impatience). For example, $\int_{t_{1}}^{t_{2}}\delta \left(x\right)\text{d}x$ is a measure of the impatience. Consequently, for an infinitesimal interval (*t*, *t* + d*t*), the measure of the impatience is given by *δ*(*t*).
3. The term *impulsivity* is used on most occasions as a synonym of impatience, but we prefer its use for intervals of the type [0, *t*] or, from an infinitesimal point of view, *δ*(0).
4. [40] use the term “self-control” as the opposite of impulsivity and therefore as a synonym of patience.

## Comparing the impatience represented by two discount functions

Most empirical studies on intertemporal choice present a set of data based on the preferences of outcomes shown by a group of individuals. The analysis of the impatience exhibited by the group is very difficult to realize because individual members of the group will show a wide variety of preferences with regard to amounts and time delays. Therefore, it is preferable to fit the resulting data to a discount function belonging to any of the noteworthy families of discount functions, viz, linear, hyperbolic, generalized hyperbolic, exponentiated hyperbolic, or exponential. The necessary adjustment can be made by using the *q*-exponential discount function (see [38] and [41]) since it includes the majority of the aforementioned functions as particular cases ([42]). Once a discount function is obtained which represents all the information coming from the individual questionnaires, it is easier to obtain the instantaneous impatience and the impatience-arc, that is to say, the impatience corresponding to a time interval. To do this, we can make use of all the tools of mathematical analysis. Moreover, the comparison between the impatience shown by two groups of people is more accurate and more easily understood, and the results can be used in designing and implementing future strategies.

### Case in which the two functions do not intersect

Let *F*_1_(*t*) and *F*_2_(*t*) be two discount functions. Assume that the ratio $\frac{F_{2}\left(t\right)}{F_{1}\left(t\right)}$ is increasing. This implies that, for every *t* > 0, $\frac{F_{2}\left(t\right)}{F_{1}\left(t\right)}>\frac{F_{2}\left(0\right)}{F_{1}\left(0\right)}=1$ and so *F*_1_(*t*) < *F*_2_(*t*). Let us recall that the patience is measured by the discount factor defined by Eq (1). As $\frac{F_{2}\left(t\right)}{F_{1}\left(t\right)}$ is increasing, for every *t*_1_ and *t*_2_ such that *t*_1_ < *t*_2_, $\frac{F_{2}\left(t_{1}\right)}{F_{1}\left(t_{1}\right)}<\frac{F_{2}\left(t_{2}\right)}{F_{1}\left(t_{2}\right)}$, from where $\frac{F_{1}\left(t_{2}\right)}{F_{1}\left(t_{1}\right)}<\frac{F_{2}\left(t_{2}\right)}{F_{2}\left(t_{1}\right)}$. Therefore,

$$\begin{array}{c} f_{1}\left(t_{1},t_{2}\right)<f_{2}\left(t_{1},t_{2}\right) \end{array}$$

and so

$$\begin{array}{c} \ln f_{1}\left(t_{1},t_{2}\right)<\ln f_{2}\left(t_{1},t_{2}\right). \end{array}$$

In particular, for every *t* and *h* > 0,

$$\begin{array}{c} \ln f_{1}\left(t,t+h\right)<\ln f_{2}\left(t,t+h\right), \end{array}$$

or equivalently

$$\begin{array}{c} \ln F_{1}\left(t+h\right)-\ln F_{1}\left(t\right)<\ln F_{2}\left(t+h\right)-\ln F_{2}\left(t\right). \end{array}$$

Therefore, if *F*(*t*) is differentiable, then

$$\begin{array}{c} {\frac{d\ln F_{1}\left(x\right)}{dx}|}_{x=t}<{\frac{d\ln F_{2}\left(x\right)}{dx}|}_{x=t}, \end{array}$$

that is to say

$$\begin{array}{c} \delta_{1}\left(t\right)>\delta_{2}\left(t\right). \end{array}$$ (3)

The converse implication is also true, whereby we can enunciate the following result.

**Theorem 2**. Let *F*_1_(*t*) and *F*_2_(*t*) be two discount functions. The following three statements are equivalent:

1. The ratio $\frac{F_{2}\left(t\right)}{F_{1}\left(t\right)}$ is increasing.
2. The impatience represented by *F*_1_(*t*) is greater than the impatience represented by *F*_2_(*t*), that is to say, *f*_1_(*t*_1_, *t*_2_) < *f*_2_(*t*_1_, *t*_2_), for every *t*_1_ and *t*_2_ such that *t*_1_ < *t*_2_.
3. If *F*_1_(*t*) and *F*_2_(*t*) are differentiable, *δ*_1_(*t*) > *δ*_2_(*t*), for every *t*.

**Example 1**. Let $F_{1}\left(t\right)=\frac{1}{1+i_{1}t}$ and $F_{2}\left(t\right)=\frac{1}{1+i_{2}t}$ be two hyperbolic discount functions where *F*_1_(*t*) < *F*_2_(*t*) (so *i*_1_ > *i*_2_). $\frac{F_{2}\left(t\right)}{F_{1}\left(t\right)}=\frac{1+i_{1}t}{1+i_{2}t}$ is increasing since

$$\begin{array}{c} \frac{d}{dt}(\frac{F_{2}}{F_{1}})\left(t\right)=\frac{i_{1}-i_{2}}{{\left(1+i_{2}t\right)}^{2}}>0. \end{array}$$ (4)

According to Theorem 2, $\delta_{1}\left(t\right)=\frac{i_{1}}{1+i_{1}t}$ must be greater than $\delta_{2}\left(t\right)=\frac{i_{2}}{1+i_{2}t}$. In effect,

$$\begin{array}{c} \delta_{1}\left(t\right)-\delta_{2}\left(t\right)=\frac{i_{1}-i_{2}}{\left(1+i_{1}t\right)\left(1+i_{2}t\right)}>0. \end{array}$$

**Fig 1:**
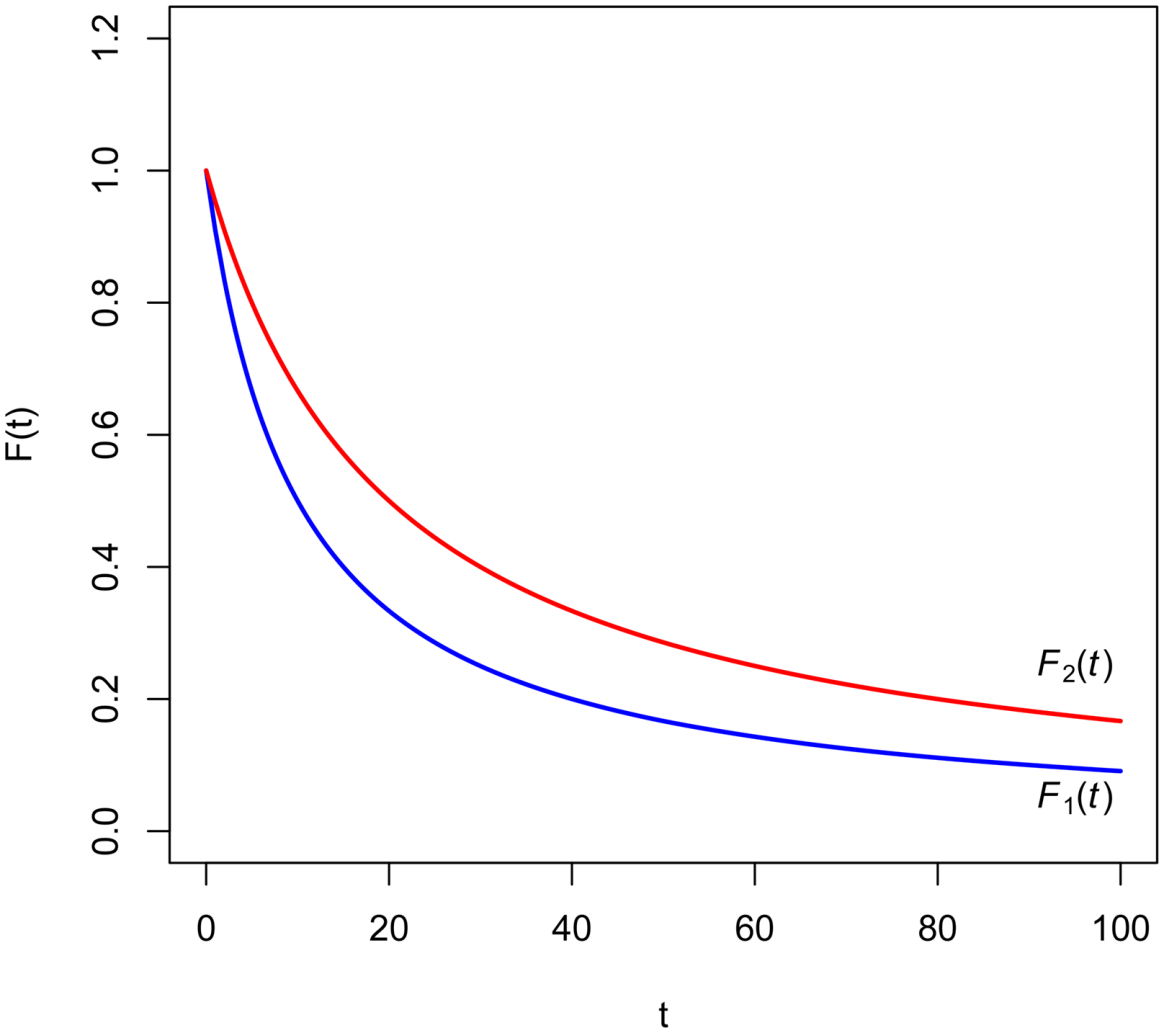
Hyperbolic discount functions of Example 1.

Fig 1 shows that it is not easy to graphically observe that the ratio *F*_2_(*t*) (shown in red) to *F*_1_(*t*) (in blue) is increasing.

For this reason we are going to formulate the following

**Corollary 1**. Let *F*_1_(*t*) and *F*_2_(*t*) be two discount functions such that *F*_2_(*t*) − *F*_1_(*t*) is increasing. In this case, any of the three equivalent conditions of Theorem 2 is satisfied. For a proof, see Appendix.

**Fig 2:**
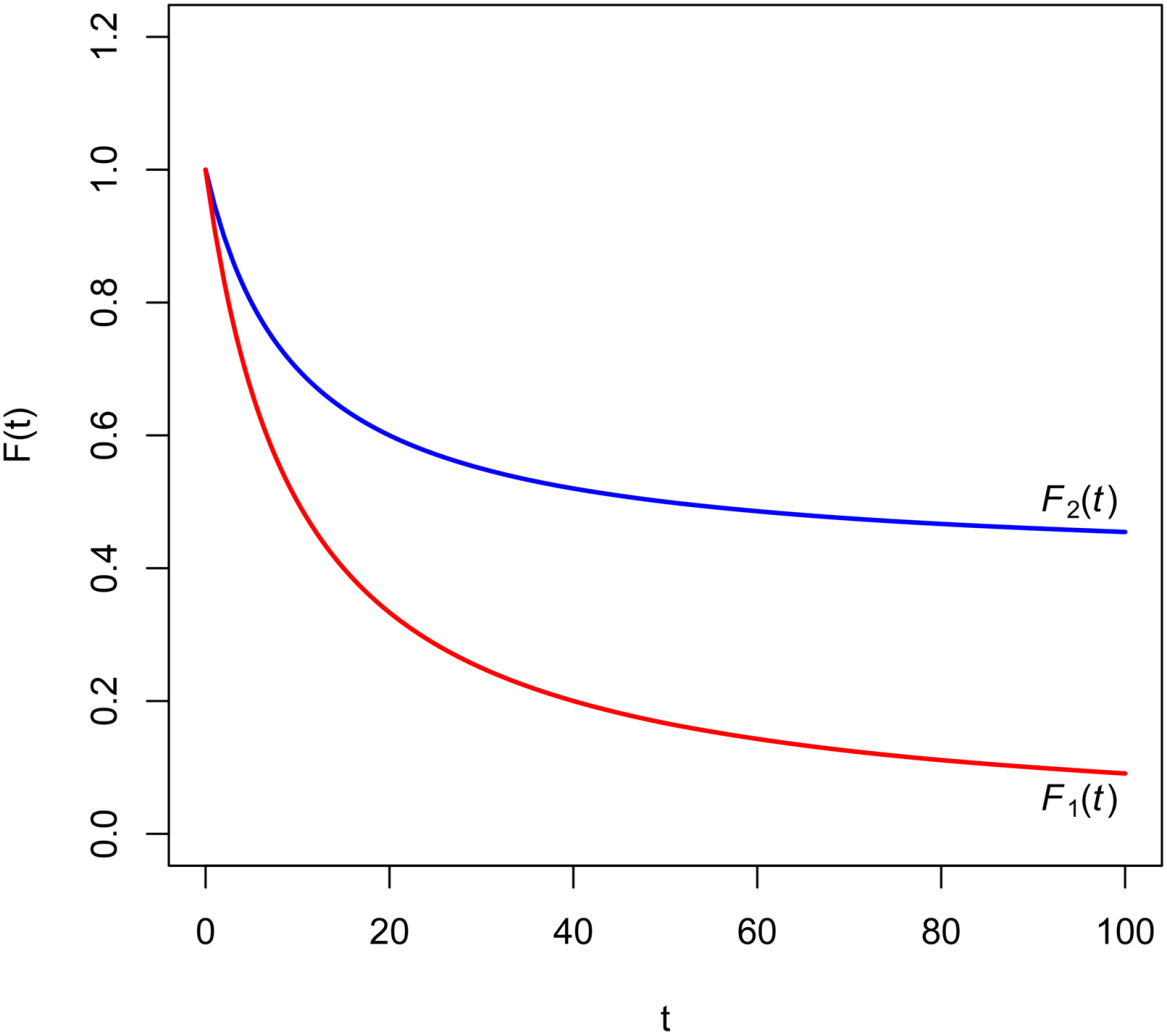
Hyperbolic discount functions of Example 2.

**Example 2**. Let $F_{1}\left(t\right)=\frac{1}{1+i_{1}t}$ be a regular hyperbolic discount function of parameter *i*_1_ and $F_{2}\left(t\right)=\frac{1+i_{2}t}{1+i_{1}t}$ be a singular hyperbolic discount function of parameters *i*_1_ and *i*_2_ (so necessarily *i*_1_ > *i*_2_). Fig 2 shows that the difference *F*_2_(*t*) − *F*_1_(*t*) is increasing.

In effect,

$$\begin{array}{c} \frac{d}{dt}\left(F_{2}-F_{1}\right)\left(t\right)=\frac{i_{2}}{{\left(1+i_{1}t\right)}^{2}}>0. \end{array}$$ (5)

**Fig 3:**
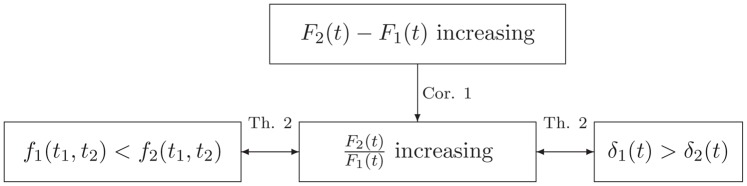
Summary of the results in Theorem 2 and Corollary 1.

According to Corollary 1, $\delta_{1}\left(t\right)=\frac{i_{1}}{1+i_{1}t}$ must be greater than $\delta_{2}\left(t\right)=\frac{i_{1}}{1+i_{1}t}-\frac{i_{2}}{1+i_{2}t}$, which can easily be verified. Then the impatience represented by *F*_1_(*t*) is greater than the impatience represented by *F*_2_(*t*). Fig 2 shows the general situation described by Corollary 1. Finally, the results obtained in Theorem 2 and Corollary 1 can be summarized in Fig 3.

**Fig 4:**
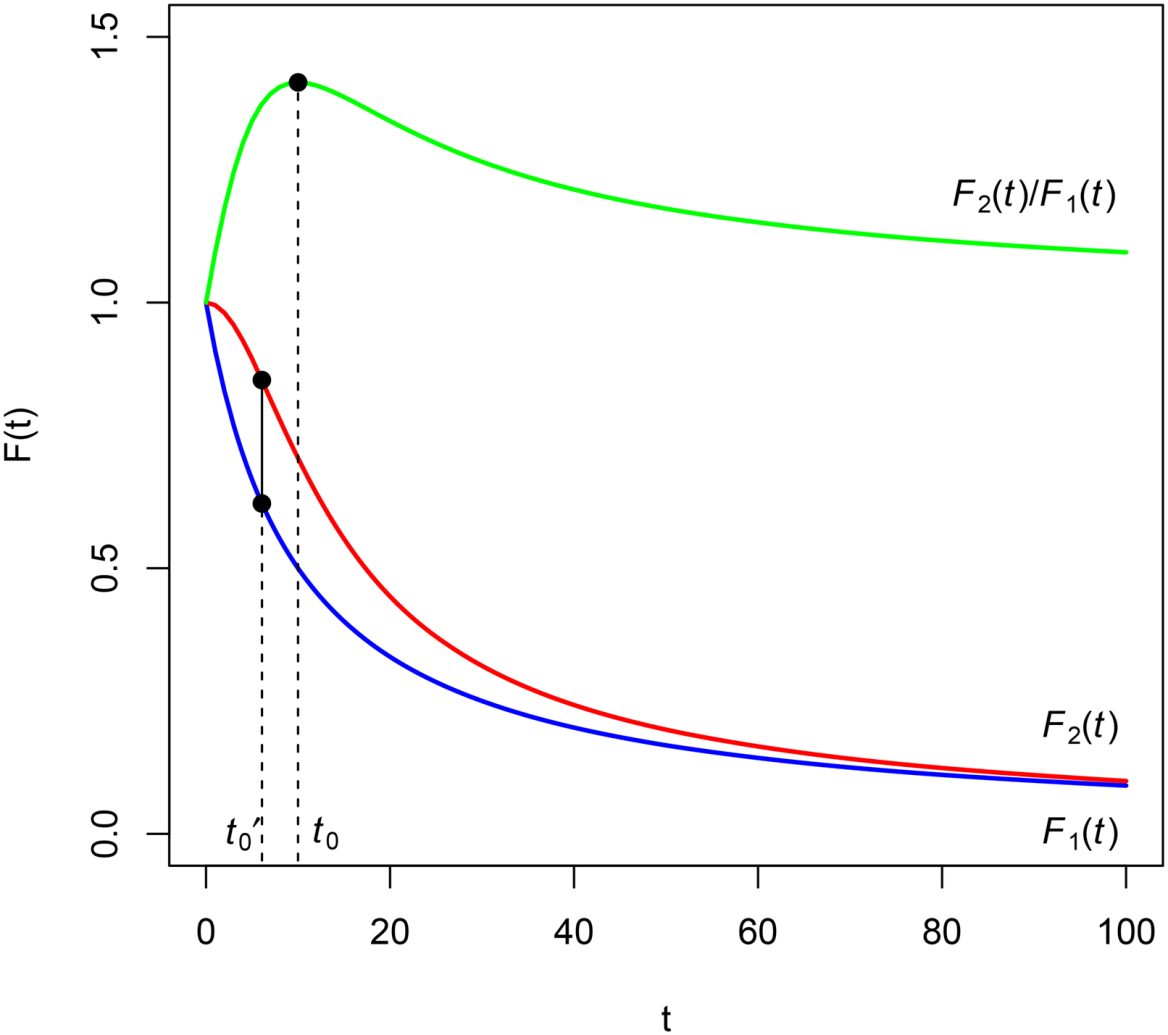
Discount functions of Example 3 and their ratio.

Let us now consider a third situation. Let us suppose that the ratio $\frac{F_{2}\left(t\right)}{F_{1}\left(t\right)}$ reaches a local maximum at instant *t*_0_. A possible graphic representation is depicted in Fig 4.

By Theorem 2, for intervals [*t*_1_, *t*_2_] included in [0, *t*_0_] (*t*_1_, *t*_2_ < *t*_0_), the impatience represented by *F*_1_(*t*) is greater than the one represented by *F*_2_(*t*). After instant *t*_0_, the opposite situation occurs, that is, the impatience represented by *F*_1_(*t*) is less than that represented by *F*_2_(*t*), but this situation can change because *t*_0_ is a local maximum and so there exists the possibility of another local extreme. For example, if *F*_2_(*t*) is singular and *F*_1_(*t*) is regular, there will exist a neighborhood of infinity where $\frac{F_{2}\left(t\right)}{F_{1}\left(t\right)}$ is increasing.

**Example 3**. Let $F_{1}\left(t\right)=\frac{1}{1+it}$ be a hyperbolic discount function of parameter *i* and $F_{2}\left(t\right)=\frac{1}{\sqrt{1+i^{2}t^{2}}}$, *i* > 0. Obviously, *F*_1_(*t*) < *F*_2_(*t*) and $\frac{F_{2}\left(t\right)}{F_{1}\left(t\right)}$ reaches a maximum at $t_{0}=\frac{1}{i}$ (see Fig 4 where *i* = 0.10). In accordance with the previous paragraph, $\delta_{1}\left(t\right)=\frac{i}{1+it}$ is greater than $\delta_{2}\left(t\right)=\frac{i^{2}t}{1+i^{2}t^{2}}$ in the interval $[0,\frac{1}{i}[=[0,10[$, and contrarily *δ*_2_(*t*) is greater than *δ*_1_(*t*) in $]\frac{1}{i},+\infty \left[=\right]10,+\infty [$.

We can now formulate the following statement.

**Theorem 3**. Let *F*_1_(*t*) and *F*_2_(*t*) be two discount functions such that *F*_1_(*t*) < *F*_2_(*t*). If *F*_2_(*t*) − *F*_1_(*t*) reaches a local maximum at $t_{0}^{\prime }$, then the factor $\frac{F_{2}\left(t\right)}{F_{1}\left(t\right)}$ reaches a local maximum at a later instant *t*_0_ (eventually, *t*_0_ can be +∞).

**Example 4**. Observe that, for the discount functions of Example 1 with *i*_1_ = 0.05 and *i*_2_ = 0.10, *F*_2_(*t*) − *F*_1_(*t*) reaches its local maximum at $t_{0}^{\prime }=12.610$ and *t*_0_ = +∞, as predicted by Theorem 3.

Table 1 schematically represents the result obtained in Theorem 3. For the sake of simplicity, we will suppose that both *F*_2_(*t*) − *F*_1_(*t*) and $\frac{F_{2}\left(t\right)}{F_{1}\left(t\right)}$ reach a unique local maximum.

**Table 1:** Several implications arising from the relationship between the local maxima of *F*_2_(*t*) − *F*_1_(*t*) and $\frac{F_{2}\left(t\right)}{F_{1}\left(t\right)}$.

**Intervals**	
$\left(0,t_{0}^{\prime }\right)$	$\left(t_{0}^{\prime },+\infty \right)$
*F*_2_(*t*) − *F*_1_(*t*) increasing	*F*_2_(*t*) − *F*_1_(*t*) decreasing
⇓ **Intervals** ⇑	
(0, *t*_0_)	(*t*_0_,+∞)
$\frac{F_{2}\left(t\right)}{F_{1}\left(t\right)}$ increasing	$\frac{F_{2}\left(t\right)}{F_{1}\left(t\right)}$ decreasing
*δ*_2_(*t*) < *δ*_1_(*t*)	*δ*_2_(*t*) > *δ*_1_(*t*)
*f*_1_(*t*_1_, *t*_2_) < *f*_2_(*t*_1_, *t*_2_)	*f*_1_(*t*_1_, *t*_2_) > *f*_2_(*t*_1_, *t*_2_)

Although *t*_0_ is the instant which separates the intervals where *δ*_1_(*t*) > *δ*_2_(*t*) and *δ*_1_(*t*) < *δ*_2_(*t*), there are some intervals [*t*_1_, *t*_2_], where *t*_1_ < *t*_0_ < *t*_2_, such that *f*_1_(*t*_1_, *t*_2_) < *f*_2_(*t*_1_, *t*_2_). In effect, given *t*_1_ < *t*_0_, this instant *t*_2_ must satisfy:

$$\begin{array}{c} \int_{t_{1}}^{t_{0}}\left[\delta_{1}\left(x\right)-\delta_{2}\left(x\right)\right]dx>\int_{t_{0}}^{t_{2}}\left[\delta_{2}\left(x\right)-\delta_{1}\left(x\right)\right]dx. \end{array}$$ (6)

The maximum value of *t*_2_ must satisfy the following equation:

$$\begin{array}{c} f_{1}\left(t_{1},t_{2}\right)=f_{2}\left(t_{1},t_{2}\right). \end{array}$$ (7)

**Fig 5:**
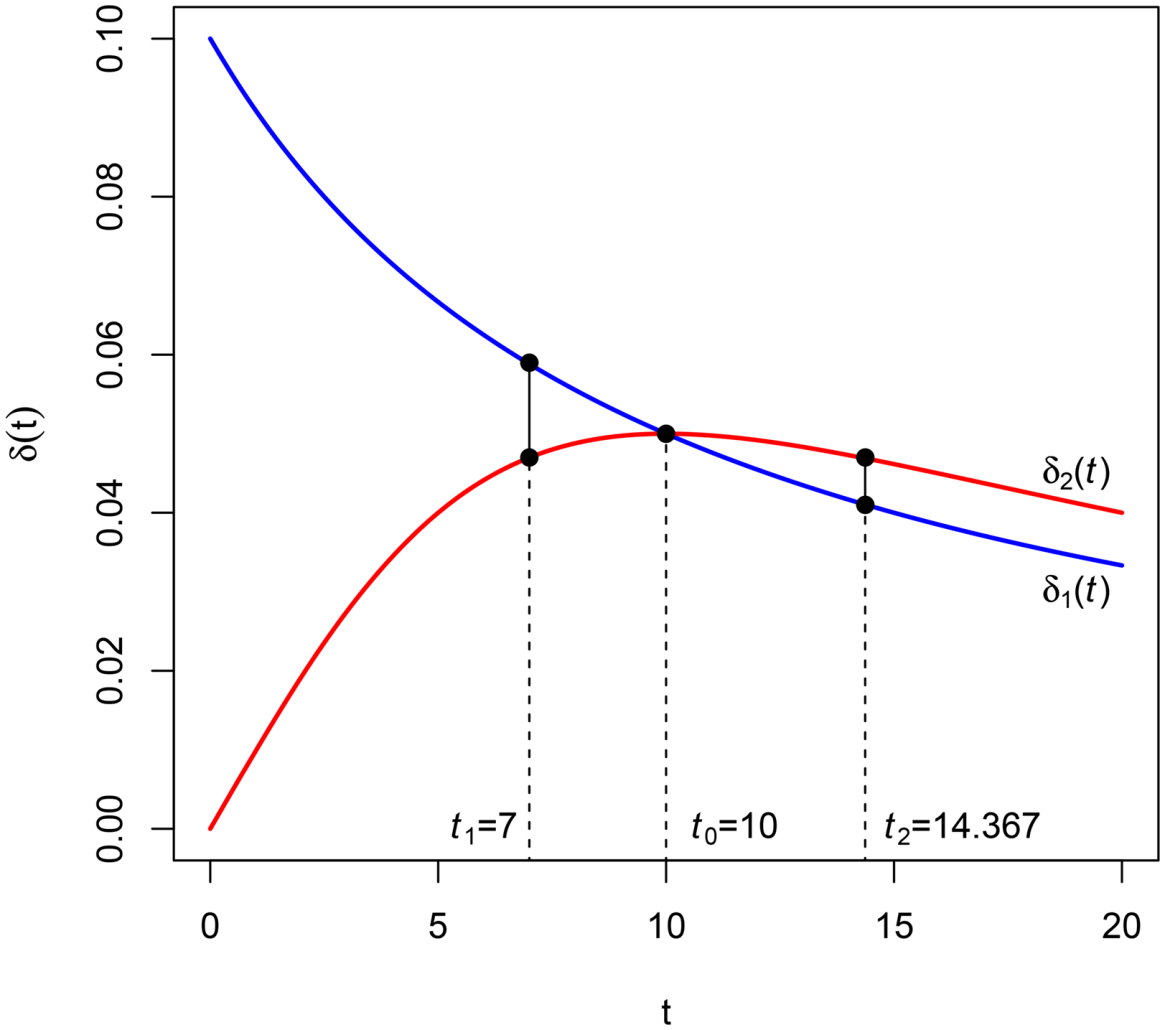
Intersection of the two instantaneous discount rates.

**Example 5**. Let *F*_1_(*t*) and *F*_2_(*t*) be the discount functions of Example 3. Taking *t*_1_ = 7, we have to solve the following equation (see Fig 5):

$$\begin{array}{c} f_{1}\left(7,t_{2}\right)=f_{2}\left(7,t_{2}\right), \end{array}$$

for which the solution is *t*_2_ = 14.367.

Finally, this reasoning can be continued by considering the following local extreme of $\frac{F_{2}\left(t\right)}{F_{1}\left(t\right)}$ (in this case, a local minimum), and so on.

### Case in which the two functions intersect

**Fig 6:**
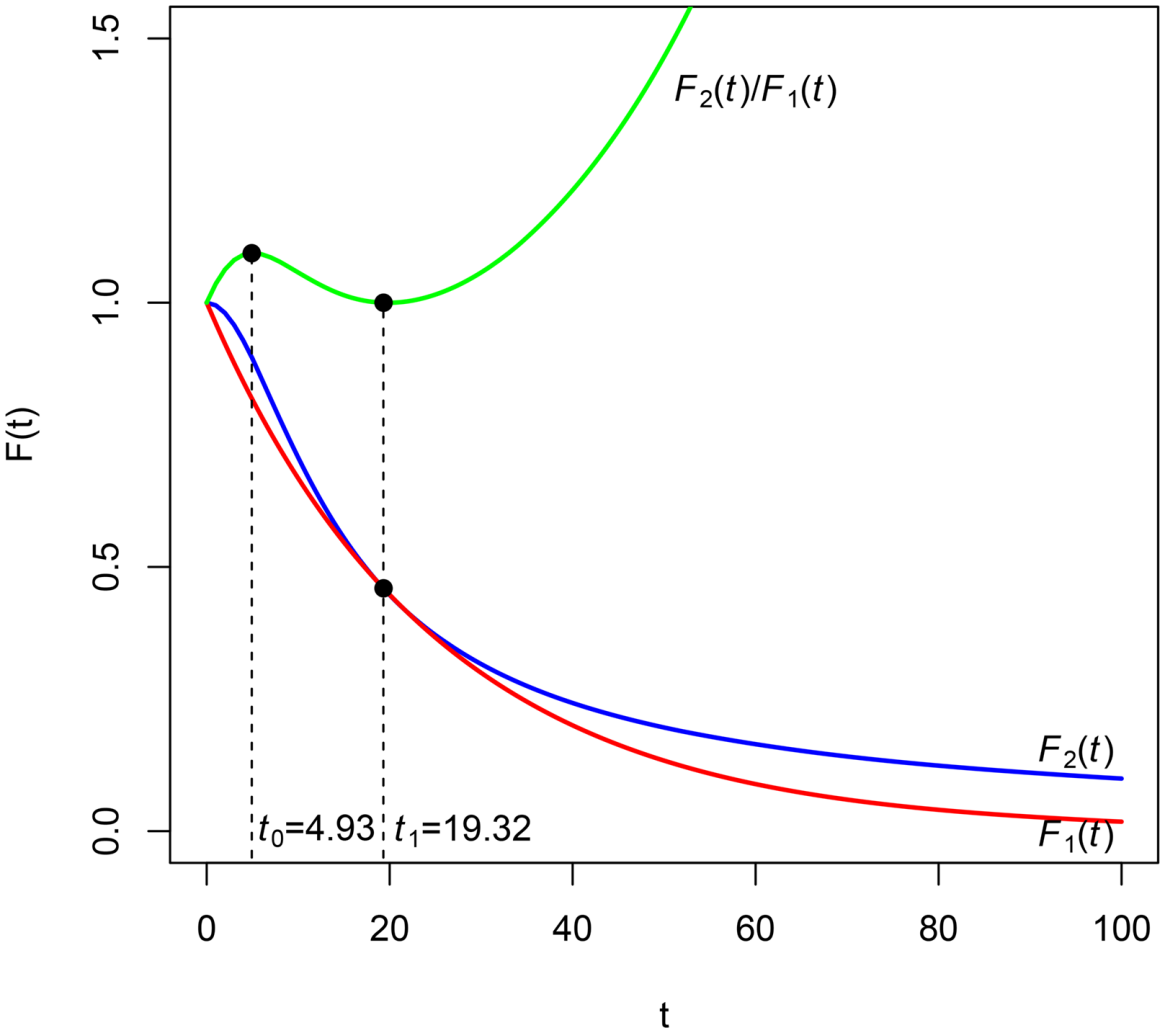
Intersection of the two discount functions: case of tangency.

For the sake of simplicity, in this Subsection, we will assume that functions *F*_1_(*t*) and *F*_2_(*t*) only intersect at an instant *t*_1_. In this case, we will distinguish between the following two subcases:

- *F*_1_(*t*) and *F*_2_(*t*) are secant. This situation does not affect the results obtained in Theorems 2 and 3.
- *F*_1_(*t*) and *F*_2_(*t*) are tangent. In this case, $\frac{F_{2}\left(t\right)}{F_{1}\left(t\right)}$ reaches a local extreme at this point and so we can apply Theorem 3. More specifically, $\frac{F_{2}\left(t\right)}{F_{1}\left(t\right)}$ reaches a local minimum at *t*_1_ (see Fig 6) and so, by Theorem 3, *δ*_1_(*t*) is less than *δ*_2_(*t*) on the left of *t*_1_, and contrarily *δ*_1_(*t*) is greater than *δ*_2_(*t*) on the right of *t*_1_. But observe also that $\frac{F_{2}\left(t\right)}{F_{1}\left(t\right)}$ reaches a local maximum at *t*_0_. Thus, the global situation can be summarized in Table 2.

**Table 2:** Patience / impatience according to different intervals.

Intervals	(0, *t*_0_)	(*t*_0_, *t*_1_)	(*t*_1_,+∞)
$\frac{F_{2}\left(t\right)}{F_{1}\left(t\right)}$	↗	↘	↗
Greater impatience	*F*_1_(*t*)	*F*_2_(*t*)	*F*_1_(*t*)
Greater patience	*F*_2_(*t*)	*F*_1_(*t*)	*F*_2_(*t*)

## An application to well-known discount functions

In experimental analysis, it is usual to fit the available data from several groups of individuals to discount functions belonging to the same family. It is therefore necessary to compare the impatience represented by two discount functions coming from the same general family.

### Comparison of two generalized hyperbolic discount functions

These functions are the well-known *q*-exponential discount functions introduced by [41]. Let *F*_1_(*t*) and *F*_2_(*t*) be two generalized hyperbolic discount functions:

$$\begin{array}{c} F_{1}\left(t\right)=\frac{1}{{\left(1+i_{1}t\right)}^{s_{1}}} \end{array}$$ (8)

and

$$\begin{array}{c} F_{2}\left(t\right)=\frac{1}{{\left(1+i_{2}t\right)}^{s_{2}}}, \end{array}$$ (9)

where *i*_1_ > *i*_2_. Let us calculate the first derivative of $\frac{F_{2}\left(t\right)}{F_{1}\left(t\right)}$:

$$\begin{array}{c} \frac{d}{dt}(\frac{F_{2}}{F_{1}})\left(t\right)=\frac{{\left(1+i_{1}t\right)}^{s_{1}-1}{\left(1+i_{2}t\right)}^{s_{2}-1}\left[s_{1}i_{1}\left(1+i_{2}t\right)-s_{2}i_{2}\left(1+i_{1}t\right)\right]}{{\left(1+i_{2}t\right)}^{2s_{2}}}. \end{array}$$ (10)

We are going to assume that *s*_1_ ≠ *s*_2_. Otherwise, the comparison between *F*_1_(*t*) and *F*_2_(*t*) would be the same as two hyperbolic discount functions. Making this derivative equal to zero, we obtain:

$$\begin{array}{c} t_{0}=\frac{s_{2}i_{2}-s_{1}i_{1}}{s_{1}-s_{2}}. \end{array}$$ (11)

1. If *s*_1_ > *s*_2_, then *t*_0_ < 0 and $\frac{F_{2}\left(t\right)}{F_{1}\left(t\right)}$ is increasing in $R^{+}$. Thus, by Theorem 2(iii), *δ*_1_(*t*) > *δ*_2_(*t*) and so the impatience represented by *F*_1_(*t*) is greater than the impatience represented by *F*_2_(*t*).
2. If *s*_1_ < *s*_2_, we can consider two subcases:
  - $s_{2}<\frac{s_{1}i_{1}}{i_{2}}$, in which case *t*_0_ > 0 is a local maximum of $\frac{F_{2}\left(t\right)}{F_{1}\left(t\right)}$. Thus, by Theorem 2, *δ*_1_(*t*) > *δ*_2_(*t*) in (0, *t*_0_) and *δ*_1_(*t*) < *δ*_2_(*t*) in (*t*_0_,+∞) and therefore, according to Theorem 2, the impatience represented by *F*_1_(*t*) is greater than the impatience represented by *F*_2_(*t*) in the interval (0, *t*_0_) and less in the interval (*t*_0_,+∞).
  - $s_{2}>\frac{s_{1}i_{1}}{i_{2}}$, in which case *t*_0_ < 0 and $\frac{F_{2}\left(t\right)}{F_{1}\left(t\right)}$ is increasing in $R^{+}$. Thus, again by Theorem 2, *δ*_1_(*t*) > *δ*_2_(*t*) and so the impatience represented by *F*_1_(*t*) is greater than the impatience represented by *F*_2_(*t*).

In order to compare the impatience of several well-known discount functions, in Table 3, we have considered the linear, hyperbolic, generalized hyperbolic, and exponential discounting both in the column on the left and on the upper row. Each cell of this table has been divided into three parts. We have represented the cases in which two discount functions *F*_1_(*t*) and *F*_2_(*t*) (*F*_1_(*t*) < *F*_2_(*t*)) satisfy the three equivalent conditions of Theorem 2. In this case, the first part of the cell shows the relationships to be satisfied by the parameters of *F*_1_(*t*) and *F*_2_(*t*) in order to satisfy Theorem 2. On the other hand, we have represented in bold those cases where *F*_1_(*t*) and *F*_2_(*t*) do not satisfy the conditions of Theorem 2. In theses cases, the first level of the cell exhibits the relationships between the parameters of *F*_1_(*t*) and *F*_2_(*t*) so that *F*_1_(*t*) < *F*_2_(*t*) in a neighborhood of zero; the second level of the cell includes the maximum *t*_0_ of $\frac{F_{2}\left(t\right)}{F_{1}\left(t\right)}$; and, finally, the third level contains the maximum $t_{0}^{\prime }$ of *F*_2_(*t*) − *F*_1_(*t*). The relative position of these time instants was discussed in Theorem 3.

**Table 3:** Cases of application of Theorem 2 or Theorem 3 (in bold), where *F*_1_ < *F*_2_.

	Discount function *F*_2_(*t*)			
Discount function *F*_1_(*t*)	Linear *F*_2_(*t*) = 1 − *d*_2_*t*	Hyperbolic $F_{2}\left(t\right)=\frac{1}{1+i_{2}t}$	Generalized hyperbolic $F_{2}\left(t\right)=\frac{1}{{\left(1+i_{2}t\right)}^{s_{2}}}$	Exponential $F_{2}\left(t\right)=\frac{1}{{\left(1+i_{2}\right)}^{t}}$
Linear *F*_1_(*t*) = 1 − *d*_1_*t*	*d*_1_ > *d*_2_	*d*_1_ > *i*_2_	*d*_1_ > *s*_2_*i*_2_	*d*_1_ > ln(1 + *i*_2_)
	–	–	–	–
	–	–	–	–
Hyperbolic $F_{1}\left(t\right)=\frac{1}{1+i_{1}t}$	***i***_**1**_ > ***d***_**2**_	*i*_1_ > *i*_2_	*i*_1_ > *s*_2_*i*_2_ and *s*_2_ < 1	***i***_**1**_ > **ln(1** + ***i***_**2**_)
	$t_{0}=\frac{i_{1}-d_{2}}{2i_{1}d_{2}}$	–	–	$t_{0}=\frac{i_{1}-\text{ln}\left(1+i_{2}\right)}{i_{1}\text{ln}\left(1+i_{2}\right)}$
	$t_{0}^{\prime }=\frac{{\left(i_{1}/d_{2}\right)}^{1/2}-1}{i_{1}}$	–	–	$\frac{{\left(1+i_{1}t_{0}^{\prime }\right)}^{2}}{{\left(1+i_{2}\right)}^{t_{0}^{\prime }}}=\frac{i_{1}}{\text{ln}(1+i_{2})}$
Generalized Hyperbolic $F_{1}\left(t\right)=\frac{1}{{\left(1+i_{1}t\right)}^{s_{1}}}$	***s***_**1**_ ***i***_**1**_ > ***d***_**2**_	*s*_1_*i*_1_ > *i*_2_ and *s*_1_ > 1	*s*_1_*i*_1_ > *s*_2_*i*_2_ and *s*_1_ > *s*_2_	***s***_**1**_ ***i***_**1**_ > **ln(1** + ***i***_**2**_)
	$t_{0}=\frac{s_{1}i_{1}-d_{2}}{i_{1}d_{2}\left(s_{1}+1\right)}$	–	–	$t_{0}=\frac{s_{1}i_{1}-\text{ln}\left(1+i_{2}\right)}{i_{1}\text{ln}\left(1+i_{2}\right)}$
	$t_{0}^{\prime }=\frac{{\left(s_{1}i_{1}/d_{2}\right)}^{1/(s_{1}+1)}-1}{i_{1}}$	–	–	$\frac{{\left(1+i_{1}t_{0}^{\prime }\right)}^{s_{1}+1}}{{\left(1+i_{2}\right)}^{t_{0}^{\prime }}}=\frac{s_{1}i_{1}}{\text{ln}(1+i_{2})}$
Exponential $F_{1}\left(t\right)=\frac{1}{{\left(1+i_{1}\right)}^{t}}$	**ln(1** + ***i***_**1**_) > ***d***_**2**_	ln(1 + *i*_1_) > *i*_2_	ln(1 + *i*_1_) > *s*_2_*i*_2_	*i*_1_ > *i*_2_
	$t_{0}=\frac{\text{ln}\left(1+i_{1}\right)-d_{2}}{d_{2}\text{ln}\left(1+i_{1}\right)}$	–	–	–
	$t_{0}^{\prime }=\frac{\text{ln ln}\left(1+i_{1}\right)-\text{ln}d_{2}}{\text{ln}(1+i_{1})}$	–	–	–

## Conclusion

The term impatience was introduced by [2] in 1930 to refer to the preference for advanced timing of future satisfaction. More recently the concept of decreasing impatience has been applied to those situations in which discount rates are decreasing. Usually this property has also been labeled as hyperbolic discounting, although there are other discount functions involving decreasing discount rates. In this paper, we have focused on measuring the degree of impatience of discount functions in both intervals and instants.

In effect, in experimental research into impatience in intertemporal choice, the data from questionnaires are usually fitted to discount functions from different families of functions. Leaving aside the problem of whether this fitting is good, once the experimental discount functions corresponding to two groups of people have been obtained, there arises the problem of comparing the impatience exhibited by each of them.

At first glance, the faster the function decreases, the higher is the degree of impatience. That is, if *F*_2_(*t*) − *F*_1_(*t*) is increasing, the impatience shown by *F*_1_(*t*) is higher than the impatience shown by *F*_2_(*t*). But this graphic criterion only represents a condition sufficient to compare degrees of impatience. Nevertheless, it is convenient to state a necessary and sufficient condition for the impatience of *F*_1_(*t*) to be higher than the impatience of *F*_2_(*t*); this condition could be that the ratio *F*_2_(*t*)/*F*_1_(*t*) is increasing. In Theorem 2, that allows us to compare the impatience associated with two discount functions, we present two conditions equivalent to the former. Unfortunately, it is difficult to observe this property graphically in most cases (unless we consider the difference ln *F*_2_(*t*) − ln *F*_1_(*t*)). In other cases, however, the monotonicity of *F*_2_(*t*)/*F*_1_(*t*) changes, necessitating the calculation of its maximum and minimum extreme values (Theorem 3). In most cases, the comparison of the impatience will be made using two discount functions belonging to the same family. Therefore, the problem of determining the local extremes of *F*_2_(*t*)/*F*_1_(*t*) can be solved explicitly or, at least, their existence must be demonstrated.

The main contributions of this paper are Theorems 2 and 3. In Table 3 we compare the impatience shown by pairs of discount functions belonging to the most important families of temporal discounting (linear, hyperbolic, generalized hyperbolic and exponential discount functions). Thus, a restriction in red represents a condition *sine qua non* for a pair of discount functions in order to satisfy Theorem 2. Nevertheless, there are other pairs of discount functions not satisfying the conditions of Theorem 2. In this case, we have deduced (as shown in bold) the expressions of *t*_0_ and (the equation to be satisfied by) $t_{0}^{\prime }$ which allows us to check the statement in Theorem 3.

These Theorems allow us to compare the impatience shown by two individuals or two groups of people, once their preferences have been fitted to a suitable discount function belonging to a well-known family of functions. Finally, this methodology can be applied to two-variable (amount and time) discount functions when some anomalies in intertemporal choice (for example, delay or magnitude effect) are taken into account.

## Appendix

*Proof of Corollary 1*. As *F*_2_(*t*) − *F*_1_(*t*) is increasing and *F*_1_(*t*) is decreasing, then $\frac{F_{2}\left(t\right)-F_{1}\left(t\right)}{F_{1}\left(t\right)}$ is increasing. Therefore, $\frac{F_{2}\left(t\right)}{F_{1}\left(t\right)}-1$ (and consequently $\frac{F_{2}\left(t\right)}{F_{1}\left(t\right)}$) is increasing which is condition (i) of Theorem 3.

*Proof of Theorem 3*. As *F*_2_(*t*) − *F*_1_(*t*) reaches a local maximum at $t_{0}^{\prime }$, then

$$\begin{array}{c} F_{2}^{\prime }\left(t_{0}^{\prime }\right)-F_{1}^{\prime }\left(t_{0}^{\prime }\right)=0, \end{array}$$ (12)

from where

$$\begin{array}{c} F_{2}^{\prime }\left(t_{0}^{\prime }\right)=F_{1}^{\prime }\left(t_{0}^{\prime }\right). \end{array}$$ (13)

As *F*_2_(*t*) > *F*_1_(*t*), and $F_{2}^{\prime }\left(t_{0}^{\prime }\right)$ and $F_{1}^{\prime }\left(t_{0}^{\prime }\right)$ are negative (remember that *F*(*t*) is decreasing), one has

$$\begin{array}{c} F_{2}^{\prime }\left(t_{0}^{\prime }\right)F_{1}\left(t_{0}^{\prime }\right)>F_{1}^{\prime }\left(t_{0}^{\prime }\right)F_{2}\left(t_{0}^{\prime }\right). \end{array}$$ (14)

Hence $F_{2}^{\prime }\left(t_{0}^{\prime }\right)F_{1}\left(t_{0}^{\prime }\right)-F_{1}^{\prime }\left(t_{0}^{\prime }\right)F_{2}\left(t_{0}^{\prime }\right)>0$ and therefore the factor $\frac{F_{2}\left(t\right)}{F_{1}\left(t\right)}$ is increasing at $t_{0}^{\prime }$, leading to a local maximum at an instant $t_{0}>t_{0}^{\prime }$ (eventually *t*_0_ could be +∞).

## References

[1] Koopmans TC (1960) Stationary ordinal utility and impatience. *Econometrica* 28(2): 287–309. 10.2307/1907722

[2] Fisher I (1930) *The Theory of Interest*. Macmillan, London.

[3] Böhm-Bawerk EV (1912) Positive Theorie des Kapitals, Dritte Auflage, English Translation in Capital and Interest, 1959, Vol. II, Positive Theory of Capital, Book IV, Section I. South Holland, pp. 257–289.

[4] Cheng J, González-Vallejo C (2014) Hyperbolic discounting: value and time processes of substance abusers and non-clinical individuals in intertemporal choice. *PLoS ONE* 9(11): e111378 10.1371/journal.pone.0111378 25390941 PMC4229090

[5] Takahashi T, Oono H, Radford MHB (2007) Empirical estimation of consistency parameter in intertemporal choice based on Tsallis’ statistics. *Physica A* 381: 338–342. 10.1016/j.physa.2007.03.038

[6] Ainslie G (1975) Specious reward: A behavioral theory of impulsiveness and impulse control. *Psychological Bulletin* LXXXII: 463–509. 10.1037/h0076860 1099599

[7] Takahashi T (2007) A comparison of intertemporal choices for oneself versus someone else based on Tsallis’ statistics. *Physica A* 385: 637–644. 10.1016/j.physa.2007.07.020

[8] Frederick S, Loewenstein G, O’Donoghue T (2002) Time discounting and time preference: a critical review. *Journal of Economic Literature* 40: 351–401. 10.1257/jel.40.2.351

[9] Cruz Rambaud S, Muñoz Torrecillas MJ (2005) Some considerations on the social discount rate. *Environmental Science and Policy* 8: 343–355. 10.1016/j.envsci.2005.04.003

[10] Cruz Rambaud S, Muñoz Torrecillas MJ (2006) Social discount rate: a revision. *Anales de Estudios Económicos y Empresariales* XVI: 75–98.

[11] Thaler R (1981) Some empirical evidence on dynamic inconsistency. *Economic Letters* 8: 201–207. 10.1016/0165-1765(81)90067-7

[12] Benzion U, Rapaport A, Yagil J (1989) Discount rates inferred from decisions: An experimental study. *Management Science* 35(3): 270–284. 10.1287/mnsc.35.3.270

[13] Takahashi T, Oono H, Radford MHB (2008) Psychophysics of time perception and intertemporal choice models. *Physica A* 387: 2066–2074. 10.1016/j.physa.2007.11.047

[14] Takahashi T, Hadzibeganovic T, Cannas SA, Makino T, Fukui H, Kitayama S (2009) Cultural neuroeconomics of intertemporal choice. *Neuroendocrinology Letters* 30(2): 185–191. 19675524

[15] Kirby K, Marakovic N (1995) Modelling myopic decisions: Evidence for hyperbolic delay-discounting within subjects and amounts. *Organizational Behavior and Human Decision Processes* 64: 22–30. 10.1006/obhd.1995.1086

[16] Myerson J, Green L (1995) Discounting of delayed rewards: Models of individual choice. *Journal of the Experimental Analysis of Behavior* 64: 263–276. 10.1901/jeab.1995.64-263 16812772 PMC1350137

[17] Kirby K (1997) Bidding on the future: Evidence against normative discounting of delayed rewards. *Journal of Experimental Psychology: General* 126: 54–70. 10.1037/0096-3445.126.1.54

[18] Takahashi T (2008) A comparison between Tsallis’ statistics-based and generalized quasi-hyperbolic discount models in humans. *Physica A* 387: 551–556. 10.1016/j.physa.2007.09.007

[19] Myerson J, Green L, Warusawitharana M (2001) Area under the curve as a measure of discounting. *Journal of the Experimental Analysis of Behavior* 76(2): 235–243. 10.1901/jeab.2001.76-235 11599641 PMC1284836

[20] Matta A, Gonçalves FL, Bizarro L (2012) Delay discounting: concepts and measures. *Psychology & Neuroscience* 5(2): 135–146. 10.3922/j.psns.2012.2.03

[21] Read D (2001) Is time-discounting hyperbolic or subadditive? *Journal of Risk and Uncertainty* 23(1): 5–32. 10.1023/A:1011198414683

[22] Prelec D (2004) Decreasing impatience: A criterion for non-stationary time preference and “hyperbolic” discounting. *Scandinavian Journal of Economics* 106(3): 511–532. 10.1111/j.0347-0520.2004.00375.x

[23] Laibson D (1997) Golden eggs and hyperbolic discounting. *The Quarterly Journal of Economics* 112(2): 443–477. 10.1162/003355397555253

[24] Halevy Y (2008) Strotz meets Allais: diminishing impatience and the certainty effect. *American Economic Review* 98(3): 1145–1162. 10.1257/aer.98.3.1145

[25] Sayman S, Öncüler A (2009) An investigation of time inconsistency. *Management Science* 55(3): 470–482. 10.1287/mnsc.1080.0942

[26] Abdellaoui M, Attema AE, Bleichrodt H (2010) Intertemporal tradeoffs for gains and losses: an experimental measurement of discounted utility. *The Economic Journal* 120(545): 845–866. 10.1111/j.1468-0297.2009.02308.x

[27] Attema AE, Bleichrodt H, Rohde KI, Wakker PP (2010) Time-tradeoff sequences for analyzing discounting and time inconsistency. *Management Science* 56(11), 2015–2030. 10.1287/mnsc.1100.1219

[28] Bleichrodt H, Rohde KI, Wakker PP (2009) Non-hyperbolic time inconsistency. *Games and Economic Behavior* 66(1): 27–38. 10.1016/j.geb.2008.05.007

[29] Tanaka T, Camerer CF, Nguyen Q (2010) Risk and time preferences: linking experimental and household survey data from Vietnam. *American Economic Review* 100(1): 557–571. 10.1257/aer.100.1.557

[30] Nguyen Q (2011) Does nurture matter: Theory and experimental investigation on the effect of working environment on risk and time preferences. *Journal of Risk and Uncertainty* 43: 245–270. 10.1007/s11166-011-9130-4

[31] Espín AM, Brañas-Garza P, Herrmann B, Gamella JF (2012) Patient and impatient punishers of free-riders. *Proceedings of the Royal Society B* 279: 4923–4928. 10.1098/rspb.2012.2043 23075842 PMC3497246

[32] Espín AM, Exadaktylos F, Herrmann B, Brañas-Garza P, (2015) Short- and long-run goals in ultimatum bargaining: impatience predicts spite-based behavior. *Frontiers in Behavioral Neuroscience* 9(214). 10.3389/fnbeh.2015.00214 PMC4538919 26347625

[33] Caliendo FN, Findley TS (2014) Discount functions and self-control problems. *Economic Letters* 122: 416–419. 10.1016/j.econlet.2013.12.035

[34] Lu Y, Zhuang X (2014) The impact of gender and working experience on intertemporal choices. *Physica A* 409: 146–153. 10.1016/j.physa.2014.05.015

[35] Chen H, Ng S, Rao AR (2005) Cultural differences in consumer impatience. *Journal of Marketing Research* XLII: 291–301. 10.1509/jmkr.2005.42.3.291

[36] Hoch SJ, Loewenstein GF (1991) Time-inconsistent preferences and consumer self-control. *Journal of Consumer Research* 17: 492–507. 10.1086/208573

[37] Seinstra M, Grzymek K, Kalenscher T (2015) Gender-specific differences in the relationship between autobiographical memory and intertemporal choice in older adults. *PLoS ONE* 10(9): e0137061 10.1371/journal.pone.0137061 26335426 PMC4559386

[38] Cruz Rambaud S, Muñoz Torrecillas MJ (2013) A generalization of the *q*-exponential discounting function. *Physica A* 392(14): 3045–3050. 10.1016/j.physa.2013.03.009

[39] Musau A (2014) Hyperbolic discount curves: a reply to Ainslie. *Theory and Decision* 76(1): 9–30. 10.1007/s11238-013-9361-8

[40] Destefano N, Martinez AS (2011) The additive property of the inconsistency degree in intertemporal decision making through the generalization of psychophysical laws. *Physica A* 390: 1763–1772. 10.1016/j.physa.2011.01.016

[41] Cajueiro DO (2006) A note on the relevance of the *q*-exponential function in the context of intertemporal choices. *Physica A* 364: 385–388. 10.1016/j.physa.2005.08.056

[42] Benhabib J, Bisin A, Schotter A (2010) Present-bias, quasi-hyperbolic discounting, and fixed costs. *Games and Economic Behavior* 69: 205–223. 10.1016/j.geb.2009.11.003

